# Bacteriological and molecular study of *Salmonella* species associated with central nervous system manifestation in chicken flocks

**DOI:** 10.14202/vetworld.2020.2183-2190

**Published:** 2020-10-20

**Authors:** Heba Badr, Mohamed A. Soliman, Soad A. Nasef

**Affiliations:** Reference Laboratory for Veterinary Quality Control on Poultry Production, Animal Health Research Institute, Agricultural Research Center, Nadi El-Seid Street, Dokki, Giza 12618, Egypt

**Keywords:** chicken, experimental study, CNS manifestations, polymerase chain reaction, *Salmonella* spp, virulence genes

## Abstract

**Background and Aim:**

*Salmonella* species often cause systemic health problems in poultry flocks, sometimes including nervous systems manifestations. This impact of *Salmonella* has rarely been studied. This study aimed to define an alternative pathogenic pathway for *Salmonella* spp. invasion of brain tissue in chicken flocks. Brain infection produces neurological manifestations; *Salmonella* strains isolated from brain tissue showed the presences of two virulence genes. Confirmation of the pathway of isolates from intestinal mucosa through the blood–brain barrier was attained using experimental infections in specific pathogen-free (SPF)-day-old chicks through two routes of inoculation.

**Materials and Methods:**

Isolation of *Salmonella* spp. from five chicken flocks that showed signs of the central nervous system (CNS) effects were isolated. Isolates were characterized by serotyping, and antimicrobial assays. In addition, virulence profiles were described using detection of virulence plasmid *spvC*, and *Salmonella* plasmid *sopB*. A pathogenicity study of isolates in specific pathogen-free (SPF)-day-old chicks through oral and intracerebral administration performed, and experimental infection in SPF embryonated chicken eggs through intra-yolk and intra-allantoic administration was investigated. Supporting histopathology and immunohistopathology against *Salmonella* antigen in brain tissue were performed for flock and experimental infections.

**Results:**

Three serotypes of *Salmonella* were isolated from the brains of five flocks (two *Salmonella* Virchow, two *Salmonella* Kentucky, and one *Salmonella* Enteritidis isolates). Phage related gene *sopB* and plasmid-mediated operon *spvC* were identified in all isolated strains. The *Salmonella* strains were re-isolated and identified from the brain and internal organs of post-experimental infected chicks. Infected chicks showed nervous manifestations associated with *Salmonella* infection. The presence of positively stained *Salmonella* antigen in brain tissues indicates penetration of the blood–brain barrier by the *Salmonella* species.

**Conclusion:**

Our results indicate that some virulent systemic strains of *Salmonella* spp. can induce CNS manifestations in chicken hosts.

## Introduction

The *Salmonella* genus includes thousands of serovars cause disease in many host species [1-3]. The genus is classified into three divisions according to host adaptation and invasiveness. Invasive serovars, such as *Salmonella* Pullorum, *Salmonella* Gallinarum in poultry, and *Salmonella* Typhi, cause human disease. The second group consists of approximately ten serovars that cause an invasive infection in poultry and may cause human infection. At present, the most important serovars are *Salmonella* Enteritidis, *Salmonella* Typhimurium*, Salmonella* Hadar, *Salmonella* Heidelberg, *S*. Saintpaul, and *Salmonella* Infantis. The third group consists of non-host-adapted and non-invasive serovars [4-9].

Non-typhoid *Salmonella* infections are associated with 2500 serovars that cause gastroenteritis in avian, bovine, and porcine hosts; the most common species *S*. Typhimurium and *S*. Enteritidis [10,11]. Intestinal disease occurs after a short incubation period of 12-72 h [12]. Limited spread of brain disease reflects an inability to overcome host defenses outside the intestinal mucosa. Still, studies in humans and mice show intermittent neurological abnormalities that permit the study of brain infection [13,14].

Mechanisms for gaining access to the central nervous system (CNS) from the intestinal mucosa by *Salmonella* are not well known, yet such invasion remains a matter of concern. An appropriate animal model is lacking, although an experiment with rabbits using *S*. Enteritidis reported meningitis [15]. Other studies have addressed pathways of transfer of intracellular pathogens to the CNS [16,17]. Modes of translocation could be intercellular, trans-cellular, cell-mediated, or other novel mechanisms [18].

Virulence plasmids are identified by a 7.8 kb region referred to as *spv* (*Salmonella* plasmid virulence), which contains five genes, designated *spv* *R, A, B, C, and D* [19]. The *spv* region promotes the rapid growth and survival of *Salmonella* spp. within the host cells, and it is important for systemic infection [20]. *Salmonella* plasmid virulence is essential for bacterial multiplication within the reticuloendothelial system of hosts [21].

The virulence of *Salmonella* spp. and their interaction with the host are complex and involve virulence factors to counter host defenses. Molecular assays for virulence genes of *S*. Enteritidis isolates from poultry in Brazil using nine genes (*lpfA*, *agfA*, *sefA*, *invA*, *hilA*, *avrA*, *sopE*, *sivH*, and *spvC*) were detected by Pilla and Tang [22]. Besides, Mezal *et al*. [23] indicate a strong distribution of *sopB* conserved genes among only a few serovars of *Salmonella* (Enteritidis, Gallinarum, and Virchow); the presence of *sopB* genes in *Salmonella* enhances invasion of epithelial cells, which may indicate their importance in pathogenesis.

Neurological manifestations induced by *Salmonella* spp. have rarely been reported in chicken flocks. *Salmonella enterica* arizonae was reported to produce neurological manifestations, with peritonitis, gastroenteritis, and hepatitis in turkey poults [24]. This study is the only published data on the issue to date**,** to the best of our knowledge.

The significance of the current study is the reinforcement of the importance of neurological disorders caused by *Salmonella* infection. This study aimed to define an alternative pathogenic pathway for *Salmonella* spp. invasion of brain tissue in chicken flocks. Brain infection produces neurological manifestations; *Salmonella* strains isolated from brain tissue showed the presences of two virulence genes. Confirmation of the pathway of isolates from intestinal mucosa through the blood–brain barrier was attained using experimental infections in specific pathogen-free (SPF)-day-old chicks through two routes of inoculation.

## Materials and Methods

### Ethical approval

Treatment of birds in experimental infections was in accordance with the regulations for the care and husbandry of experimental animals and approved by the Animal Care Committee of the Animal Health Research Institute, Giza, Egypt.

### Study period and location

The study was performed between November 2017 and December 2019 at the Reference Laboratory for Veterinary Quality Control on Poultry Production.

### Collection of samples

Brain samples were aseptically collected from freshly dead or euthanized diseased broiler chickens from five Cobb flocks. Birds selected showed CNS manifestations–lying down with stretched legs, ataxia, and infrequent tremor. Age of infected birds varied from 1 to 34 days.

### Isolation and identification of *Salmonella* spp.

Bacteria were isolated and identified, as previously described [25]. Briefly, pre-enrichment of brain tissue used Buffer Peptone Water (Oxoid^®^, UK) incubated at 37°C for 16-18 h. One-tenth mL of pre-enrichment medium was transferred to Modified Semisolid Rappaport-Vassiliadis medium (LabM^®^, UK) and incubated at 41.5°C for 24 h. Furthermore, 1 mL was transferred to MKTTn broth (LabM^®^, UK) and incubated aerobically for 24 h. The samples were then streaked onto XLD (LabM^®^, UK) and SS (Oxoid^®^, UK) agar plates and incubated at 37°C for 24 h, aerobically. Typical colonies were identified by biochemical tests (Urea agar, Triple sugar iron, and Lysin iron) (Oxoid^®^, UK).

### Serotyping of isolated *Salmonella* spp.

Serotyping was accomplished as previously described [26], followed by the reading of *Salmonella* species using the Kauffman–White scheme [27] with *Salmonella* antisera (Sifin^®^, Japan).

### Antimicrobial sensitivity test for *Salmonella* strains

The antibiogram of *Salmonella* isolates used disk-diffusion tests as previously described [28] against 17 antibiotics (Oxoid^®^, UK) (ampicillin, chloramphenicol, ciprofloxacin, clindamycin, colistin sulfate, doxycycline, florfenicol, fosfomycin, gentamicin, levofloxacin, lincomycin, nalidixic acid, neomycin, norfloxacin, streptomycin, tetracycline, and trimethoprim-sulfamethoxazole). Assay results were interpreted according to CLSI/NCCLS [29].

### Molecular detection of virulence genes *sopB* and *spvC*

A conventional polymerase chain reaction (PCR) assay was performed for detection of virulence genes. DNA was extracted from isolates using a QIAamp DNA Mini Kit following kit instructions, (Qiagen, Germany, GmbH) catalog no. 51304. PCR used extracted DNA using specific primers (Metabion, Germany) [30,31] to amplify virulence genes (*sopB* and *spvC*) (Table-1) using PCR Master Mix (Takara, Japan). The amplification was performed in a Biometra T3 thermal cycler. The PCR products were separated by electrophoresis on 1% agarose gel according to Sambrook *et al*. [32] (Applichem, Germany) in 1× TBE buffer at room temperature (24°C) using a gradient of 5 V/cm. Fifteen μL of PCR product was loaded in each gel slot. A 100 bp DNA Ladder (Qiagen, Germany) was used to determine fragment sizes. The gel was photographed with a gel documentation system (Alpha Innotech, Biometra, Germany).

**Table 1:** Primers sequence, target genes, amplified sizes, and annealing condition.

Target gene	Primers sequences	Amplified product	Annealing	References
*sopB*	F- TCAGAAGRCGTCTAACCACTC	517 bp	58°C	[30]
	R- TACCGTCCTCATGCACACTC		40 s	
*spvC*	F- ACCAGAGACATTGCCTTCC	467 bp	58°C	[31]
	R- TTCTGATCGCCGCTATTCG		40 s	

### Experimental study

#### Preparation of bacterial inoculums

Five *Salmonella* serotypes (two isolates *S*. Virchow, two *S*. Kentucky, and one *S*. Enteritidis*)* were prepared according to Osman *et al*. [33] from frozen stocks by sub-culturing into Buffered peptone water ( Oxoid^®,^, UK) and incubating at 37°C for 24 h aerobically. A loopful was streaked onto XLD agar plate (LabM^®^, UK) and incubated overnight at 37°C, aerobically. A colony was then inoculated into Tryptic Soy Broth (TSB, Merck^®^, Germany) and incubated overnight at 37°C aerobically. The overnight culture in TSB was serially diluted in sterile normal saline to reach an inoculum of 10^6^ CFU/ml for oral dosing and 10^4^ (for intracerebral and embryonated chicken eggs [ECE]). CFU estimates were confirmed by colony counting on Tryptic Soya Agar plates.

#### Pathogenicity study on one-day-old chicks

The experiment was designed to examine the pathogenicity of five *Salmonella* isolates in 110 SPF chicks – ten chicks for each group (Table-2). Two routes of inoculation (oral and intracerebral) were used. The chicks were divided into 11 groups of ten chicks each: One control group, and two trial groups for each isolate, one for oral, and one for intracerebral inoculation). Ten SPF day-old chicks per group were housed in separate controlled biosafety cages at the RLQP experiment animal house. A day before infection samples were collected (pooled fecal samples and internal organs of 1-day-old chicks) and tested to confirm the absence of *Salmonella*. Birds were kept off feed for 12 h before inoculation to reduce crop bulk, thus speeding flushing of inocula.

**Table 2:** Groups of Pathogenicity study on 1-day-old chicks.

Serotypes	Number of groups	Number of SPF chicks (orally)	Number of SPF chicks (intra cerebral)	Negative control
*Salmonella* Virchow	Gr. No. 1	10	10	10
	Gr. No. 2	10	10	
*Salmonella* Kentucky	Gr. No. 3	10	10	
	Gr. No. 4	10	10	
*Salmonella* Enteritidis	Gr. No. 5	10	10	
Total=110		50	50	10

#### Oral pathogenicity

Infection dose was 0.1 mL introduced orally for each animal in each oral exposure group (1×10^5^ CFU/mL) for observation of morbidity and mortality. Birds were observed for 15 days. Birds had access to feed and water *ad libitum* with 24 h of light daily according to Osman *et al*. [33]. Euthanasia of SPF chicks and re-isolation of *Salmonella* from internal organs and brain were performed.

#### Intracerebral pathogenicity

SPF-day-old chicks were inoculated intracerebrally with 0.1 mL (final concentration 1×10^3^ CFU/mL) according to Bryan and Scheld [15] to monitor strain pathogenicity. Later, re-isolation of *Salmonella* serotypes from the brain and internal organs was used to assess transmission through the blood–brain barrier. The level of pathogenicity of an isolate was estimated by monitoring clinical signs and mortality rate.

#### Inoculation of SPF fertile eggs

One hundred and ten SPF ECE were candled for intra-yolk inoculation of 1×10^3^ CFU/mL at day 6 (50 eggs), intra-allantoic at 11 day (50 eggs), and ten SPF eggs were used as negative control; each of the five *Salmonella* strains was inoculated twice into 10 SPF eggs with 0.1 mL of 1×10^4^ CFU/mL of bacterial culture (intra-yolk and intra-allantoic routes). The results were used to identify transmission *Salmonella* serotypes from parent, and persistence of such infection through hatching.

#### Immunohistopathology

Chicken brains were collected and fixed in 10% formalin. Following dehydration, tissues were embedded in paraffin and sections using a microtome. Sections were deparaffinized in xylene and rehydrated through a series of decreasing alcohol solutions and finally immersed in distilled water for 5 min. Some sections were stained with hematoxylin-eosin [34], while others were processed for staining with antibody against *Salmonella* somatic O antigen using a primary rabbit anti-*Salmonella* antibody (ab35156) and Secondary-Goat Anti-Rabbit IgG H&L (HRP) (ab205718). Staining with DAB brown was used to visualize bacterial antigen microscopically [35].

## Results

### Isolation, identification, and serogrouping

Examination of collected brain samples for *Salmonella* isolation identified five isolates of *Salmonella* spp. Serotypes for these isolates were *S*. Virchow, two strains (O 6, 7, 14; r; 1, 2), *S*. Kentucky, two strains (O 8, 20; i; Z_6_), and *S*. Enteritidis, one strain (O1, 9, 12; g, m;--).

#### Antimicrobial sensitivity test for *Salmonella* strains

All strains were resistant to clindamycin and lincomycin while, four of five were resistant to ampicillin, gentamycin, nalidixic acid, streptomycin, tetracycline, and trimethoprim-sulfamethoxazole and three of five were resistant to doxycycline and norfloxacin. In contrast, all strains were highly susceptible to colistin sulfate and fosfomycin and three of five were sensitive to ciprofloxacin and levofloxacin (Table-3).

**Table 3:** Results of antimicrobial sensitivity tests.

Antimicrobial discs	*Salmonella* spp*.* isolates interpretation (n=5)		

	Sensitivity (%)	Intermediate (%)	Resistance (%)
Ampicillin	1 (20)	0	4 (80)
Chloramphenicol	2 (40)	1 (20)	2 (40)
Ciprofloxacin	3 (60)	0	2 (40)
Clindamycin	0	0	5 (100)
Colistin sulfate	5 (100)	0	0
Doxycycline	1 (20)	1 (20)	3 (60)
Florfenicol	2 (40)	1 (20)	2 (40)
Fosfomycin	5 (100)	0	0
Gentamicin	1 (20)	0	4 (80)
Levofloxacin	3 (60)	2 (40)	0
Lincomycin	0	0	5 (100)
Nalidixic acid	1 (20)	0	4 (80)
Neomycin	1 (20)	2 (40)	2 (40)
Norfloxacin	2 (40)	0	3 (60)
Streptomycin	1 (20)	0	4 (80)
Tetracycline	1 (20)	0	4 (80)
Trimethoprim-sulfamethoxazole	1 (20)	0	4 (80)

### Molecular detection of virulence genes

Isolated strains of *Salmonella* all showed the presence of phage related gene *sopB* and the plasmid-mediated operon *spvC* (Figure-1a and b).

**Figure-1a:**
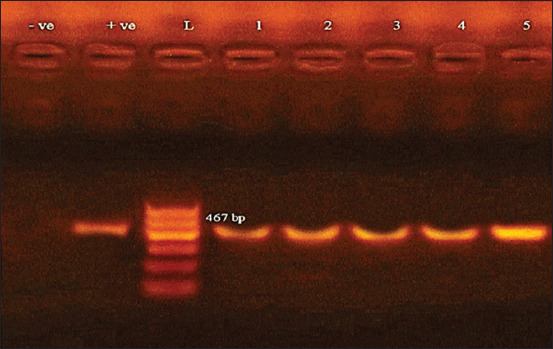
Agarose gel electrophoresis showing amplification of 467 bp fragments using PCR was performed with primer specific for *spvC* gene. Lane (1-5) shows the positive amplification of five isolates. L: Ladder (100-600). +ve: Positive control and –ve: Negative control.

**Figure-1b:**
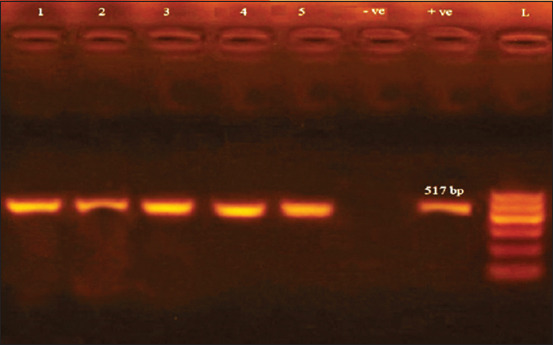
Agarose gel electrophoresis showing amplification of 517 bp fragments using PCR was performed with primer specific for *sopB* gene. Lane (1-5) shows the positive amplification of five isolates. L: Ladder (100-600). +ve: Positive control and –ve: Negative control.

### Pathogenicity study

SPF-day-old chicks were inoculated by two routes. Chicks administered bacteria orally showed different mortality and morbidity rates. Chicks inoculated with *S*. Virchow showed 40% mortality in one group and 0% in the second group. However, chicks in both groups exhibited diarrhea after 1 week of infection along with closed eye and difficulty breathing. The two groups of chicks inoculated with *S*. Kentucky showed 20% mortality and the same signs as chick inoculated with *S*. Virchow. No mortality was seen in chicks inoculated with *S*. Enteritidis; these chicks again showed diarrhea, closed eye, and difficulty breathing.

Chicks administered by intracerebral injection showed 100% mortality before 24 h. Chicks struggled and stretched legs and wings before death. Severe congestion of blood vessels of skull and internal organs, with omphalitis, was noted (Table-4).

**Table 4:** Results of *Salmonella* isolates from bacteriological examination from collected brain tissue and mortality rate in pathogenicity study.

Serotypes/(Number of isolates)	Number of group	Mortality rate by oral route % (Number of dead/total)	Mortality rate in intra-cerebral route % (Number of dead/total)
*Salmonella* Virchow (2)	Gr. No. 1	40 (4/10)	100 (10/10)
	Gr. No. 2	0 (0/10)	100 (10/10)
*Salmonella* Kentucky (2)	Gr. No. 3	20 (2/10)	100 (10/10)
	Gr. No. 4	20 (2/10)	100 (10/10)
*Salmonella* Enteritidis (1)	Gr. No. 5	0 (0/10)	100 (10/10)

After 2 weeks postmortem examination of euthanized surviving chicks from oral route inoculation showed congestion of muscle and internal organs, ballooning of intestine–especially ceca–unabsorbed yolk sac, and congestion of the skull region and, in varying degrees, of the brain. The re-isolation of *Salmonella* from organs, intestine, and brain was successful for all groups of chicks and for all serovars.

SPF ECE were also inoculated through two routes. Intra-yolk inoculation at 6 days of age caused 100% mortality after 24 h for one *S*. Kentucky serovar, while other serovars (two *S*. Virchow, one *S*. Kentucky, and one *S*. Enteritidis) showed 100% mortality 5 days after inoculation. Congestion of the embryo and yolk sac was found on examination of dead embryos.

Intra-allantoic inoculation of *S*. Virchow at 11 days of age showed 50% mortality after 24 h, 90% after 3 days, and 100% after 10 days. *S*. Kentuck*y* showed 100% mortality after 3 days. *S*. Enteritidis caused 100% mortality after 24 h. severe congestion in embryo and yolk sac was observed on examination of dead embryos. The re-isolation of *Salmonella* from embryo and yolk sacs was successful for all strains.

#### Histopathology

Histopathological examination showed focal mononuclear cell aggregations in cerebral tissue in naturally infected birds (Figure-2a). Necrotic foci with cellular debris, surrounded by macrophages, vacuolation and microgliosis, were observed after oral route inoculation with *S*. Virchow (Figure-2b).

**Figure-2a:**
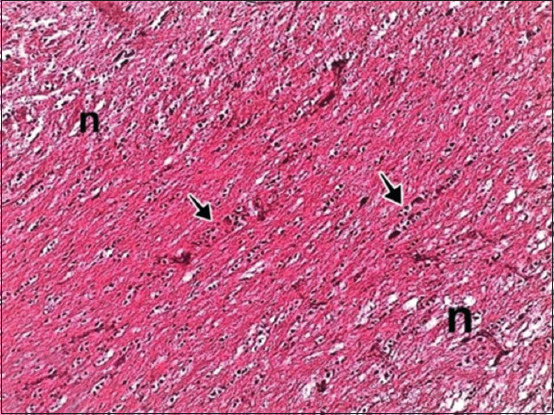
Brain showed focal necrosis (n) with accumulation of mononuclear cells (arrow) H and E, 200×.

**Figure-2b:**
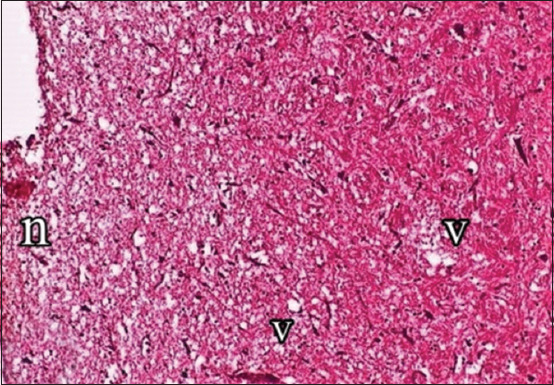
Brain showed focal necrosis (n) with vacuolation (v) in cerebrum. H and E, 200×.

#### Immunohistopathology

**Figure-3:**
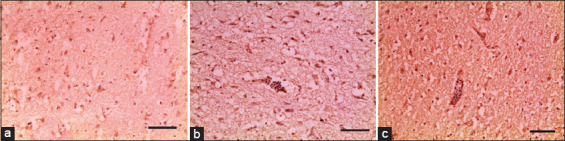
Brain sections showed positive reaction of *Salmonella* LPS antigen of dark brown spots in natural infected chickens (a), in intracerebral route of experimental infected chickens (b) and in oral route (c), scale bar 200μm.

Antibodies specific to *Salmonella* O antigen showed the presence of positively stained bacterial antigen in the cortex and medulla, consistent with findings in naturally infected chickens (Figure-3a)**,** in experimental intracerebral infection with *S*. Virchow (Figure-3b) and in oral infection with *S*. Virchow (Figure-3c).

## Discussion

*Salmonella* is a relatively common hazard in developing countries, though seldom seen in developed countries [36,37]. Brain infection is an unusual consequence of salmonellosis [38]. In many species of animals, *Salmonella* meningitis is a major cause of death and brain damage and is correlated with a particularly poor prognosis [39,40]. *Salmonella* spp. are the most common cause of death attributed to bacterial meningitis [41].

We seldom find studies on *Salmonella* isolated from poultry brain. *Salmonella* spp. is the leading cause of bacterial meningitis in children, account for almost 13% of childhood bacterial meningitis cases [42,43]. Isolated *Salmonella* from meningitis cases is serotyped as *S*. Typhimurium, *S*. Enteritidis, *S*. Panama, *S*. Bredeney, *S*. Infantis, *S*. Virchow, *S*. Sandiego, and *S*. Newport [44]. A report from the Centers for Disease Control cited by Wilson and Feldman [45] indicates that *S*. Heidelberg and *S*. Saintpaul accounted for most salmonellae isolated from the cerebrospinal fluid of patients in the USA.

In the current study, bacterial isolation from collected brain samples of broilers suffering from CNS manifestation at age ~15 days old showed five isolates of *Salmonella* spp. Two isolates were identified and serotyped as *S*. Virchow, two isolates *S*. Kentucky, and one isolate of *S*. Enteritidis. Other cases show evidence of *Salmonella* meningitis in several species, but not in poultry [46,47]. However, *Salmonella* isolation was absent in brains of examined broiler chicken flocks in the Kafr El- Sheikh Province in northern Egypt [48].

Antimicrobial therapy is an important tool in reducing the enormous losses in the poultry industry caused by *Salmonella* infections. However, resistance to existing antimicrobials is widespread and of concern to poultry veterinarians [49]. Administration of antimicrobials to chickens at therapeutic and sub-therapeutic levels has been an integral part of poultry production, and this practice plays a role in encouraging antibiotic-resistant organisms. Once established, resistant organisms can spread from farm to humans through consumption of contaminated food [50]. Antimicrobial drug use in livestock production is implicated in the development and dissemination of drug resistance to public health [51,52]. Examination of five *Salmonella* strains for antibiotic resistance revealed high resistance in all strains to several widely used drugs and two or three of the of five strains were resistant to doxycycline and norfloxacin to most of the remaining antibacterials. High sensitivity in all strains was found only for colistin sulfate and fosfomycin among tested agents**.**

Mendonça *et al*. [53], found low levels of resistance for sulfonamides (75.8%) and nitrofurantoin (52.8%), tetracycline (15.4%), streptomycin (7.7%), nalidixic acid (7.7%), gentamicin (5.5%), norfloxacin (3.3%), trimethoprim (3.3%), cefalotin (2.2%), ampicillin (1.1%), and chloramphenicol (1.1%). Tolerance to ciprofloxacin was not observed, suggesting a slow progression to resistance. This problem raises concerned among scientists that *Salmonella* meningitis would see a high rate of medication failure, a high rate of relapse, and significant neurological sequelae Herold *et al*. [54]. In the current study, the application of molecular identification of virulence genes for isolated strains that all strains showed the presence of phage related gene *sopB* and the plasmid-mediated operon *spvC*. Connor *et al*. [55] showed that the presence of the *sopB* gene in isolated strains of *S*. Enteritidis and *S*. Typhimurium has zoonotic potential.

The *sopB* gene is encoded in SPI-1 and is identified in isolates involved in major epidemics; *sopB* has therefore been identified as a key to the emergence of epidemic strains [56]. All *S*. Enteritidis isolated in another study from different species, human, chicken, and egg houses, report positive results for *sopB*. This factor is likely to be critical for pathogenesis [57].

Orally inoculated in day-old-SPF chicks in our study showed different mortality (0-40%) with different morbidity rates. Intracerebral inoculation showed 100% mortality for all *Salmonella* serotypes before 24 h. Chick struggled with stretched legs and wings before death. Re-isolation of *Salmonella* from the lungs, stomach, and brain from all groups of chicks shows that salmonellae had passed through the blood–brain barrier, from organs to the brain and vice versa.

Whether *Salmonella* invades the CNS primarily as intracellular bacteria is not known [58]. Pathogenesis is affected not only by the dosage and route of inoculation but also by the genetic background and immune state of the host [59,60].

 However, inoculation of SPF embryonic chicken eggs through intra-yolk and intra-allantoic injection caused 100% mortality at times ranging from 24 h to 5 days. All ECE infections with isolated *Salmonella* spp. induced lack of hatchability.

To the best of our knowledge, studies on infection with *Salmonella* in the chicken brain are limited, but *Salmonella* spp. are extensively studied in mice. An important role for *Salmonella* pathogenicity island (SPI) 1 and outer membrane protein A genes enable blood–brain barrier penetration [14]. Furthermore, behavioral abnormalities in infected mice show neurological signs of illness (twirling, and loss of motor coordination) associated with numerous bacteria in brain, spleen, and liver. Others demonstrated that oral infection of mice with *S. enterica* serovar Typhimurium resulted in meningitis and brain infection [61].

Limited studies that record CNS manifestation in turkey poults due to *Salmonella enterica arizonae* infection [24] associated with gastroenteritis and hepatitis. We conclude that both presence of *sopB* and *spvC* virulent genes for isolated *Salmonella* strains and positive reaction obtained from the use of specific antibodies to *Salmonella* LPS antigen in neuronal tissue provide strong evidence of the invasiveness of isolated pathogenic *Salmonella*. Further, results highlight that some *Salmonella* spp. serovars cross the blood–brain barrier that is considered an alternative pathogenic pathway.

## Conclusion

We conclude that certain serovars of *Salmonella* spp. have the ability to induce adverse CNS signs in chickens after crossing the blood–brain barrier. Further, investigation is needed using other serovars to confirm the alternative infection pathway.

## Authors’ Contributions

HB and SAN designed the study. HB and MAS performed the experiments, processed the experimental data and performed the validation analysis. HB and MAS analyzed the results and drafted the manuscript. HB and MAS revised and finalized the manuscript for submission. All authors read and approved the final manuscript.
